# Diagnostic accuracy and prognostic significance of Glypican-3 in hepatocellular carcinoma: A systematic review and meta-analysis

**DOI:** 10.3389/fonc.2022.1012418

**Published:** 2022-09-23

**Authors:** Donglei Jiang, Yingshi Zhang, Yinuo Wang, Fu Xu, Jun Liang, Weining Wang

**Affiliations:** ^1^ Department of General Surgery, Grand Hospital of Shuozhou, Shuozhou, China; ^2^ Department of Clinical Pharmacy, Shenyang Pharmaceutical University, Shenyang, China

**Keywords:** Glypican-3, hepatocellular carcinoma, diagnosis, prognosis, meta-analysis

## Abstract

**Purpose:**

Glypican-3 (GPC-3) expression is abnormal in the occurrence and development of hepatocellular carcinoma (HCC). To explore whether GPC-3 has diagnostic accuracy and prognostic significance of HCC, we did a systematic review and meta-analysis.

**Method:**

PubMed, Embase, Cochrane Library, and China National Knowledge Infrastructure were searched with keywords “GPC-3” and “HCC” and their MeSH terms from inception to July 2022. We applied the hierarchical summary receiver operating characteristic model and evaluated the diagnostic value of GPC-3 alone and combination, and the correlation between high and low GPC-3 expression on clinicopathological features and survival data in prognosis.

**Results:**

Forty-one original publications with 6,305 participants were included, with 25 of them providing data for diagnostic value and 18 records were eligible for providing prognostic value of GPC-3. GPC-3 alone got good diagnostic value in patients with HCC when compared with healthy control and moderate diagnostic value when compared with patients with cirrhosis. In addition, combination of GPC-3 + AFP and GPC-3 + GP73 got great diagnostic value in HCC versus cirrhosis groups; the combination of GPC-3 can also improve the diagnostic accuracy of biomarkers. Moreover, we discovered that overexpression of GPC-3 was more likely found in HBV infection, late tumor stage, and microvascular invasion groups and causes shorter overall survival and disease free survival, which means poor prognosis.

**Conclusion:**

GCP-3 could be used as a biomarker in HCC diagnosis and prognosis, especially in evaluated diagnostic value in combination with AFP or GP73, and in forecasting worse survival data of overexpression GPC-3

**Systematic Review Registration:**

https://www.crd.york.ac.uk/PROSPERO/, identifier [CRD42022351566].

## Introduction

Hepatocellular carcinoma (HCC) is a kind of high-degree malignancy, with incidence rate and mortality rising year over year, and ranking sixth and fourth respectively in the world (1). HCC onset was hidden in the early stage, which can only be found in imaging scans (2–4). In addition, the malignancy develops rapidly in the middle to late stage, leading to the poor prognosis of patients with HCC. Moreover, for patients with cirrhosis, the risk of progression to HCC is high, so early diagnosis of HCC, especially in patients with cirrhosis, is particularly important (5–9). At present, alpha-fetoprotein (AFP), the most commonly used tumor marker in HCC, has a poor diagnostic performance (10, 11). Hence, it is particularly important to improve the diagnostic value of early HCC, especially HCC in cirrhosis. Recent studies have found that Glypican-3 (GPC-3) expression is abnormal in the occurrence and development of HCC (12, 13), which may be related to the diagnosis and prognosis of HCC.

GPC-3 protein is a heparan sulfate glycoprotein on the cell membrane surface, which is connected to the cell membrane surface through glycosylphosphatidylinositol anchor (14). GPC-3 anchored to the cell surface is a key glycoprotein that interacts with cells and extracellular matrix components. It has a strong potential to bind to functional biological macromolecules such as proteins and sugars, especially to a variety of cell growth factors, including vascular endothelial growth factor, epidermal growth factor, hepatocyte growth factor, fibroblast growth factor, and transforming growth factor–β. GPC-3 binds to a variety of growth factors to form receptor signal transduction complexes, which activate the tyrosine kinase activity of growth factor receptors, thereby regulating cell growth, differentiation, adhesion, proliferation, and migration, which may be related to the occurrence and progression of HCC (15–18).

However, the diagnostic accuracy and prognostic significance of GPC-3 have not yet been determined. The aim of our meta-analysis was to explore the role of GPC-3 in the diagnostic accuracy and prognostic significance of HCC versus controls. None of the published meta-analyses (19–21) provide a comprehensive overview of the diagnostic and prognostic roles of GPC-3 in tumorigenesis.

## Methods

The selected publications and synthesis program followed the Predesigned Reporting Items for Systematic Reviews and Meta-analyses (PRISMA) statement for diagnostic test accuracy and prognostic test significance (22, 23). In addition, the protocol of our study were pre-designed and registered with the PROSPERO website (No. CRD42022351566) (24).

### Search strategy and selection criteria

Four online electronic databases (PubMed, Embase, Cochrane Library, and China National Knowledge Infrastructure) were searched using keywords “Glypican-3”, “GPC-3”, “Hepatocellular Carcinoma”, “Liver cancer”, and “HCC” and their MeSH terms from their inception to July 2022. Two researchers (DJ and YZ) independently screened titles, abstracts, and full text. Moreover, the disagreements were discussed with the sophisticated reviewer (WW).

Eligible studies should meet the following criteria: research studies should focus on the diagnostic test accuracy and/or prognostic test significance of GPC-3, and patients should be diagnosed with HCC, with useful data available. For diagnostic type study, both data from HCC group and non-HCC group were considered to evaluate the diagnostic value of GPC-3. For prognostic type study, the data of correlation between high and low GPC-3 expression were considered. No language restriction was set, and non-English articles were translated. Reviews and original studies with no control group and with a study population being patients with HCC recurrence were excluded. Moreover, the reference list of previously published articles was also checked to avoid omissions of potential articles.

### Data extraction and quality assessment

Baseline characteristic and clinical diagnostic and prognostic data were extracted from every potential included research studies and were categorized into pre-set forms by two independent researchers (DJ and YZ). For studies that reported data of GPC-3 diagnostic accuracy, baseline characteristic of the first author, publication year, region, sample type, control type, detection method, cutoff value of GPC-3, sample size, gender, age, hepatitis B virus (HBV) infection (present/absent), hepatitis C virus (HCV) infection (present/absent), cirrhosis (present/absent), Child–Pugh score (B–C vs. A), and diagnostic value data of true positive (TP), false negative (FN), true negative (TN), and false positive (FP) were also extracted for assessment.

For studies that reported data of GPC-3 prognostic significance, baseline characteristic of the first author, publication year, region, detection method, cutoff value of GPC-3, sample size, gender, age, clinicopathological features of tumor size (small vs. big), HBV (present/absent), HCV (present/absent), cirrhosis (present/absent), AFP(<20 ng/ml/>20 ng/ml), tumor grade (I–II/III–IV), microvascular invasion (present/absent), Child–Pugh score (A vs. B–C), BCLC grade (A–B vs. C–D), differentiation (Well-moderate vs. high), and survival data of overall survival (OS) rate (high expression vs. low expression) and disease-free survival (DFS, high expression vs. low expression) in high versus low expression of GPC-3 were also extracted for assessment.

In diagnostic studies for the quality assessment process, modified Quality Assessment of Diagnostic Accuracy Studies 2 (QUADAS-2) tool was used (25). QUADAS-2 consists of four domains: patient selection, index test, reference standard, and flow of patients through the study, which could be included without high-risk options. None of the above domains could contain high-risk options; otherwise, the original research will not be allowed to be included in this meta-analysis. In prognostic studies for the quality assessment process, the Newcastle–Ottawa Scale (NOS) scale (26) was used. If a study got NOS score of less than 4 (max 10), the observational study then cannot be included in this meta-analysis.

In addition, the quality of evidence for both types of research was assessed on the basis of the GRADE system. For diagnostic outcomes, grades for recommendations, assessments, developments, and evaluations were estimated (27). In addition, for prognostic study, the risk of bias, imprecision, inconsistency, indirectness of the every outcome, and publication bias were evaluated (28). Moreover, quality assessment programs were also independently assessed by two independent researchers.

### Outcomes and data synthesis

To pool outcomes from diagnostic research, we applied the hierarchical summary receiver operating characteristic (ROC) model (29) and evaluated the diagnostic value of overexpression GPC-3, GPC-3 + AFP, GPC-3 + GP73, and GPC-3 + GP73 + AFP as biomarkers under investigation. Moreover, sensitivity, specificity, positive likelihood ratio, negative likelihood ratio, diagnostic odds ratio, and their 95% confidence intervals (CIs) as well as the area under the ROC curves were obtained from diagnostic models of overall participants. Subgroup analysis and meta-regression for each subtype of control include cirrhosis and hepatitis, cirrhosis only, and healthy control. A P-value less than 0.05 from the meta-regression indicates that this grouping method had a great impact on the overall results. Moreover, Deeks’ asymmetry test was used to determine potential publication bias.

To merge outcomes from prognostic research, random-effects models were applied to consider significant difference from odds ratios (ORs), hazard ratio (HR), or standardized mean differences (SMDs) with their 95% CIs in both clinicopathological features and survival data. *I* ^2^ > 50% or *P*-value < 0.05 indicates high heterogeneity. Moreover, Begg’s and Egger’s tests were used to determine the publication bias among the included studies (30, 31). MetaDisc (version 1.4) and STATAMP (version 14.0) software were used to perform this meta-analysis.Results

## Results

### Study characteristics and quality assessment

**Figure 1:**
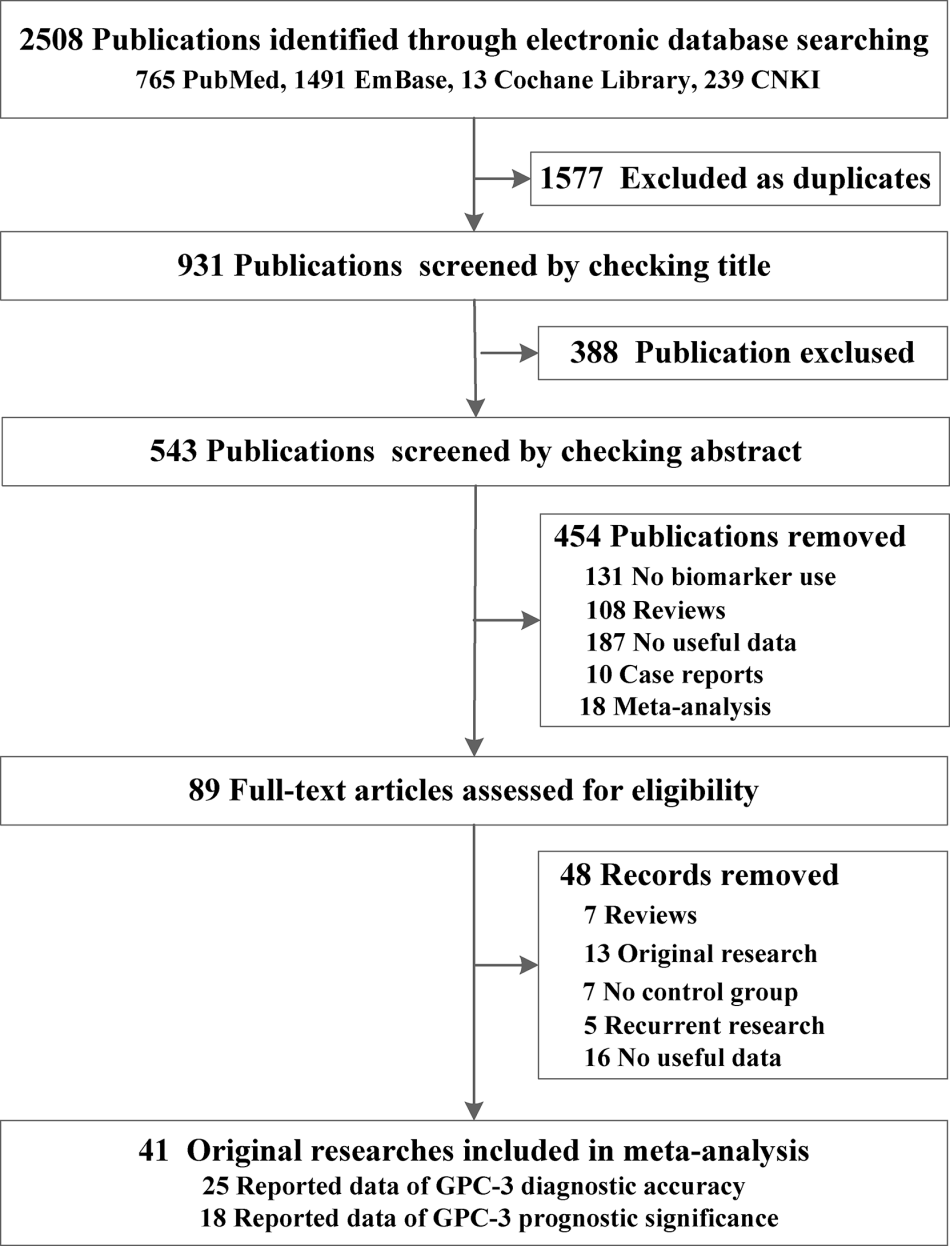
Preferred reporting items for systematic reviews and meta-analyses flowchart of the identification of eligible research studies.

From the four online electronic databases, 931 non-repetitive publications have been screened. After checking titles and abstracts, 89 full-text articles have been assessed for eligibility. After careful screening, 40 articles (31–72) could be included in this meta-analysis, 25 of them (3,717 participants) reported data of GPC-3 diagnostic accuracy and 18 of them (2,588 patients with HCC) reported data of GPC-3 prognostic significance (**Figure 1**). The sample size of our included studies was 22–261, and most of them were published in Asia. We also summarized the control type, indicator type, detection method, and cutoff value; the cutoff value varied widely among the included studies that could have great influence on the overall results (**Table 1**; see details in **Tables S1**, **S2**). Moreover, we also meta-analyzed baseline indicator to determine whether the baseline is balance. We could notice that from diagnostic type baseline, there are more male patients with HCC, older, and more patients with HCC with liver cirrhosis and worse liver function grades (Child–Pugh score B–C). Moreover, baseline characteristic was balanced in data from prognostic significance studies (**Table 1**). Moreover, the quality assessment of the included studies is presented in **Tables S3**, **S4**; all of them got acceptable score.

**Table 1:** Summarized of baseline characteristic of the included research studies.

Diagnostic value of GPC-3 high expression				
Control type	Detection method	Cutoff value	Study (year)	Sample size
Non-HCC	Serum, IHC	–	Coral et al., 2021 (34)	156
	Serum, ELISA	>498.7 pg/ml	Tan, 2020 (41)	62
	Serum, ELISA	>4.9 pg/ml	Farag et al., 2019 (42)	250
	Tissue, IHC	>10%	Uthamalingam et al., 2018 (47)	22
	Serum, ELISA	>6 pg/ml	Attallah et al., 2016 (49)	256
	Serum, ELISA	>0.38 ng/ml	Zhu et al., 2016 (52)	261
	Tissue, IHC	>400 ng/L	Yan et al., 2015 (56)	116
	Tissue, IHC	>25%	Liu et al., 2014 (59)	182
	Serum, ELISA	>60 pg/ml	Ma et al., 2014 (60)	218
	Serum, ELISA	>805.38 pg/ml	Long et al., 2013 (63)	172
	Tissue, IHC	>3 score	Wang et al., 2011 (69)	268
	Tissue, WB	–	Liu et al., 2009 (70)	65
Cirrhosis and hepatitis	Serum, ELISA	>5 ng/ml	Cao et al., 2021 (32)	200
	Serum, ELISA	>64 pg/ml	Caviglia et al., 2021 (33)	191
	Serum, ELISA	>0.057 ng/ml	Malov et al., 2021 (34)	110
	Serum, CLEIA	>73 pg/ml	Caviglia et al., 2020 (39)	349
	Serum, ELISA	> 1.2 ng/ml	Gomaa et al., 2020 (40)	60
	Serum, ELISA	> 0.850 pg/ml	Li et al., 2019 (43)	136
	Serum, ELISA	> 6.595 pg/ml	Tahon et al., 2019 (44)	70
	Serum, ELISA	> 5.8 µg/L	Unić et al., 2018 (46)	70
	Serum, ELISA	6 pg/ml	Attallah et al., 2016 (49)	200
	Serum, WB	>15 ng/ml	Yang et al., 2013 (64)	77
Healthy control	Serum, ELISA	> 1.2 ng/ml	Gomaa et al., 2020 (40)	50
	Serum, ELISA	–	El-Saadany et al., 2018 (45)	100
	Serum, ELISA	> 8.98 μg/L	Fu et al., 2013 (62)	90
	Serum, ELISA	> 6.08 pg/ml	Li et al., 2014 (58)	82
	Serum, ELISA,IHC	>120 ng/ml	Song et al., 2011 (68)	104
**Baseline characteristic**				
Indicator	OR/SMD^a^ (95% CI)	*P*, *I* ^2^	Balance or not	
Gender	1.538 (1.122, 2.107)	0.000, 60.5%	Not	
Age^a^	0.576 (0.491, 0.660)	0.000, 90.3%	Not	
HBV (Present/Absent)	0.589 (0.240, 1.445)	0.026, 72.7%	Yes	
HCV (Present/Absent)	2.238 (0.411, 12.170)	0.000, 94.2%	Yes	
Cirrhosis (Present/Absent)	3.622 (1.636, 8.015)	0.114, 59.9%	Not	
Child–Pugh score (B–C vs. A)	1.818 (1.033, 3.201)	0.475,0.0%	Not	
**Prognostic value of GPC-3 high expression**				
**Indicator type**	**Detection method**	**Cutoff value**	**Study (year)**	**Sample size**
Clinicopathological features and survival data	Tissue, IHC	>5%	Jeon et al., 2016 (50)	185
	Tissue, IHC	>33%	Wang et al., 2016 (51)	135
	Tissue, IHC	>10%	Cui et al., 2015 (53)	104
	Serum, IHC	>2 score	Haruyama et al., 2015 (54)	115
	Tissue, IHC	>10%	Pan et al., 2015 (55)	300
	IHC	>6 score	Ning et al., 2012 (65)	61
Clinicopathological features	Immunoreactivity	≥ 5%	Zhao et al., 2021 (37)	143
	Serum, qRT⁃PCR	≥1/3	Zhou et al., 2021 (38)	126
	Tissue, IHC	>30%	Xue et al., 2017 (48)	316
	Tissue, IHC	>25%	Liu et al., 2014 (59)	101
	Tissue, IHC	>9 score	Fan et al., 2013 (61)	35
	Tissue, IHC	>25%	Fu et al., 2013 (62)	160
	Tissue, IHC	>10%	Wang et al., 2012 (66)	31
	Tissue, IHC	>30%	Yu et al.et al., 2012 (67)	316
	Tissue, IHC	>3 score	Wang et al., 2011 (69)	114
	WB	–	Li et al., 2006 (71)	41
	Tissue, IHC	–	Ding et al., 2005 (72)	41
Survival data	–	–	Wang et al., 2021 (36)	264
**Baseline characteristic**				
Indicator	OR/SMD^a^ (95% CI)	*P*, *I* ^2^	Balance or not	
Gender	0.952 (0.713, 1.272)	0.514, 0.0%	Yes	
Age	1.031 (0.743, 1.430)	0.544, 0.0%	Yes	
Age^a^	−0.159 (−0.332, 0.015)	0.221, 30%	Yes	

IHC, immunohistochemistry.

### Diagnostic value of GPC-3

**Figure 2:**
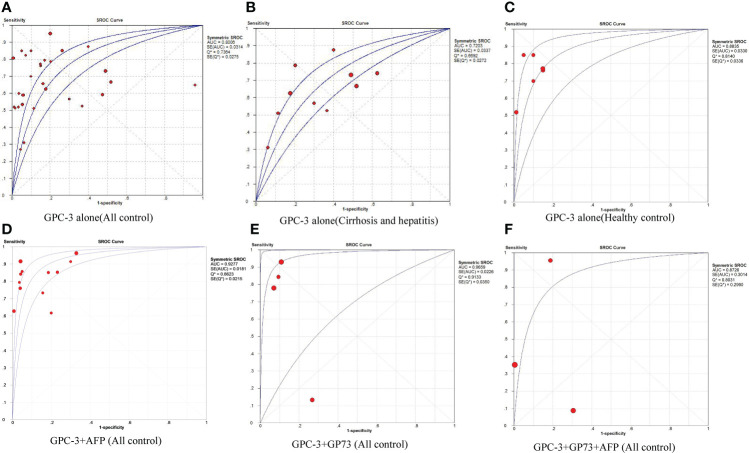
The pooled diagnostic accuracy of overexpression of GPC-3 alone in diagnosing HCC vs. all controls **(A)**, HCC vs. cirrhosis and hepatitis **(B)**, HCC vs. healthy control **(C)**, and combination diagnostic accuracy of GPC-3 + AFP **(D)**, GPC-3 + GP73 **(E)**, and GPC-3 + GP73 + AFP **(F)** in diagnosing HCC vs. all controls. AUC, area under the curve; GPC-3, Glypican-3; HCC, hepatocellular carcinoma; SROC, summary receiver operating characteristic curves.

First, we meta-analyzed data for the diagnostic value of GPC-3 alone in HCC. We can notice that the diagnostic value of GPC-3 alone in HCC vs. all control type is good with AUC of 0.8006 (**Figure 2A**), and the good diagnostic value could also be found in HCC vs. healthy control subgroup (AUC = 0.8835; **Figure 2C**). Nonetheless, the diagnostic value of HCC vs. cirrhosis and hepatitis/cirrhosis alone was ordinary, with AUC = 0.7203 and 0.7326 (**Figure 2B**). Meta-regression did not declare the main source of the difference. No publication bias was found from Deeks’ asymmetry test in the meta-analysis of GPC-3’s diagnostic value, and the quality of evidence was low to moderate. These results demonstrate that GPC-3 alone has good diagnostic efficacy in patients with HCC compared with healthy people (**Table 2**; **Figure S1**). However, for patients with liver cirrhosis progressing to HCC, its diagnostic efficiency is not high, so improving the diagnostic efficiency in this population has more clinical value.

**Table 2:** Summarized of pooled sensitivity, specificity, positive likelihood ratio, negative likelihood ratio, diagnostic odds ratio, and SROC curve of GPC-3 in diagnosing hepatocellular carcinoma patients from controls.

Marker	Control type	No. of studies (participants)	Sensitivity	Specificity	Positive likelihood ratio	Negative likelihood ratio	Diagnostic odds ratio	AUC from SROC Curve	P-value from meta-regression	Publication bias	Grade
GPC-3 alone	All controls	25 (3,931)	0.69 (0.66, 0.71)	0.76 (0.74, 0.78)	4.35 (2.99, 6.32)	0.41 (0.34, 0.50)	12.19 (6.96, 21.36)	0.8006*	0.6984	0.968	Moderate
	Cirrhosis and hepatitis	9 (1,435)	0.66 (0.63, 0.70)	0.62 (0.59, 0.66)	2.03 (1.52, 2.71)	0.56 (0.45, 0.66)	4.15 (2.53, 6.82)	0.7203		0.324	Moderate
	Cirrhosis only	7 (1,298)	0.69 (0.65, 0.73)	0.61 (0.57, 0.64)	1.97 (1.45, 2.70)	0.52 (0.42, 0.65)	4.18 (2.36, 7.37)	0.7326		0.320	Moderate
	Healthy control	5 (446)	0.74 (0.68, 0.79)	0.90 (0.85, 0.94)	6.27 (4.07, 9.67)	0.28 (0.19, 0.41)	26.26 (14.46, 47.67)	0.8835*		0.628	Low
GPC-3 + AFP	All controls	11 (1,480)	0.91 (0.77, 0.98)	0.70 (0.84, 0.89)	7.70 (4.33, 13.69)	0.20 (0.14, 0.29)	46.45 (22.89, 94.26)	0.9277**	0.6006	0.031^#^	Moderate
	Cirrhosis and hepatitis	2 (260)	0.73 (0.65, 0.81)	0.82 (0.74, 0.88)	4.08 (2.76, 6.03)	0.32 (0.18, 0.55)	13.20 (5.89, 29.59)	0.8883*		0.988	Low
	Healthy control	2 (186)	0.85 (0.76, 0.91)	0.95 (0.89, 0.99)	18.41 (7.05, 48.13)	0.16 (0.10, 0.25)	117.11 (37.16, 369.02)	0.500		–	Low
GP73 + GPC-3	All controls	4 (511)	0.72 (0.65, 0.79)	0.87 (0.83, 0.90)	4.68 (1.35, 16.23)	0.25 (0.04, 1.84)	18.18 (1.36, 243.47)	0.9659**		0.514	Low
AFP + GP73 + GPC-3	All controls	3 (365)	0.50 (0.41, 0.59)	0.87 (0.82, 0.91)	3.90 (0.30, 50.67)	0.49 (0.18, 1.31)	13.07 (0.13, 1,345.16)	0.8726*		0.848	Low

*Significant differences; ^#^Publication bias. **Better diagnostic value.

Then, to improve the diagnostic value of HCC compared with liver cirrhosis, we studied the combined diagnostic value of GCP-3 combination with AFP and GP73. In combination diagnostics value of GPC-3 + AFP, great diagnostics value could be found in HCC vs. all control type of AUC of 0.9277 (**Figure 2D**). However, publication bias could also be found in this group of 0.031 and moderate GRADE. In addition, great diagnostics value could be found in HCC vs. cirrhosis and hepatitis, with AUC of 0.8883 with low grade; the value of HCC vs. healthy control is low due to small sample size. Subsequently, great diagnostics value could also be found in HCC vs. all control group in GPC-3 + GP73 (AUC = 0.9659; **Figure 2E**) and GPC-3 + GP73 + AFP (AUC = 0.8726; **Figure 2F**) diagnostic groups (**Table 2**; **Figure S1**). In general, the diagnostic efficacy of GPC-3 combined with AFP or GP73 has been improved; especially, GPC-3+ AFP group can improve the diagnostic value of HCC in patients with liver cirrhosis.

### Prognostic value of GPC-3

First, we summarized the clinicopathological features of high GPC-3 vs. low GPC-3 expression in patients with HCC. We could notice that significant differences could be found in HBV (present/absent) group (OR = 1.340; 95% CI: 1.015 to 1.768) with low heterogeneity (*P* = 0.465, *I* ^2^ = 0.0%), tumor grade (III–IV/I–II) group (1.653, 1.201 to 2.276) with low heterogeneity (1.653, 1.201 to 2.276), and microvascular invasion (present/absent) group (1.830, 1.009 to 3.318) with substantial heterogeneity (0.011, 59.7%). No publication bias was found in all clinicopathological features with low to moderate grade. These results indicate that high GPC-3 expression is not good for the prognosis of patients with HCC, especially patients with HCC with HBV infection, late tumor stage, and microvascular invasion.

**Figure 3:**
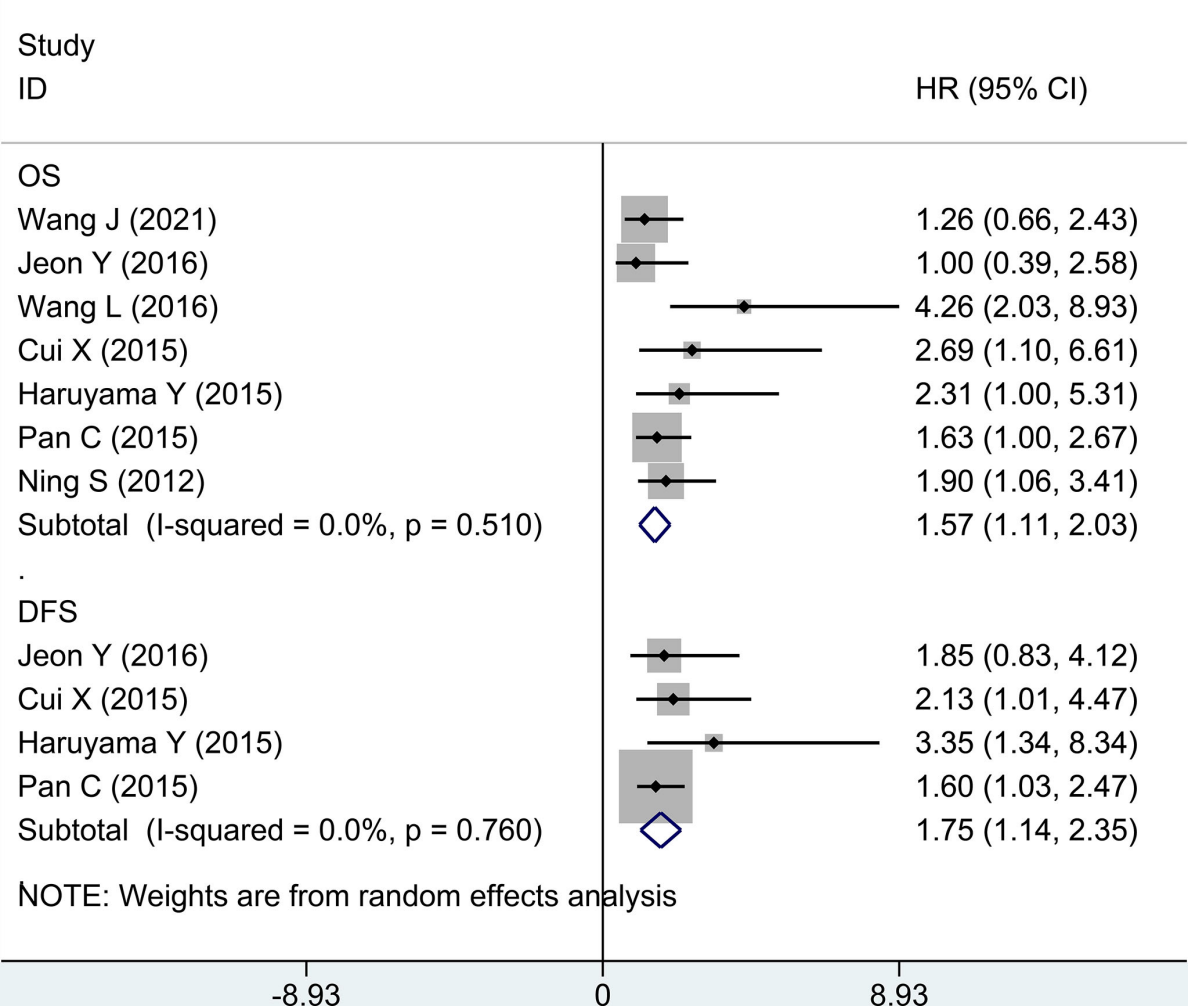
Forest plot for high GPC-3 expression versus low GPC-3 expression for overall survival and disease-free survival in patients with HCC. GPC-3, Glypican-3.

Second, in evaluating survival data of high expression vs. low expression of GPC-3 in HCC, OS and DFS were taken into account. In terms of OS, seven research studies have reported ordinary data, and the merged outcome was (HR = 1.57; 95% CI: 1.11 to 2.03) with low heterogeneity (*P* = 0.510, *I* ^2^ = 0.0%). When considering DFS, only four studies provide data, and the outcome from meta-analysis was (1.75, 1.14 to 2.35; 0.760, 0.0%) (**Figure 3**). The above data prove that the high expression of GPC-3 has a poor prognosis for patients with HCC.

## Discussion

This work focuses on the diagnostic accuracy and prognostic significance of GPC-3 as a biomarker of HCC, with 41 original publications and 6,315 participants included. First, we did a meta-analysis to explore whether the baseline level is balanced or not. The baseline indicators were not balanced in gender, age, liver cirrhosis numbers, and Child–Pugh liver function in diagnostic accuracy research studies (**Figure 1**; **Table 1**). Second, we did a diagnostic meta-analysis to evaluate the diagnostic value of GPC-3 alone. We determine that, compared with healthy control, the diagnostic value of GPC-3 alone is relatively great. However, in comparison with liver cirrhosis, the diagnostic value of GPC-3 alone is moderate. As a result, combination diagnostic value of GPC-3 was researched, and we detected that combination use of GPC-3 + AFP and GPC-3 + GP73 group got great diagnostic value of AUC > 0.9 in HCC versus cirrhosis groups (**Figure 2**; **Table 2**). These results demonstrate that the combination of GPC-3 can also improve the diagnostic accuracy of tumor biomarkers that have already been used in clinical practice (e.g., AFP). Third, the clinicopathological features and survival data in prognosis of HCC were meta-analyzed. We discovered that overexpression of GPC-3 was more likely found in HBV infection, late tumor stage, and microvascular invasion groups and causes shorter OS and DFS, which means poor prognosis (**Figure 3**; **Table 3**).

**Table 3:** Summarized of association between high GPC-3 expression and clinicopathological features in prognosis of patients with hepatocellular carcinoma from low GPC-3 expression.

Clinicopathological features	No. of studies (participants)	OR (95% CI)	Heterogeneity test	Significance	Publication bias	Grade
Tumor size (Small vs. Big)	11 (1,197)	1.524 (0.959, 2.422)	0.017, 53.7%^#^	No	0.876, 0.706	Low
HBV (Present/Absent)	12 (1,923)	1.340 (1.015, 1.768)*	0.465, 0.0%	Yes	1.000, 0.425	Moderate
HCV (Present/Absent)	2 (300)	0.442 (0.186, 1.049)	0.239, 27.8%	No	1.000,-	Low
Cirrhosis (Present/Absent)	10 (1,769)	1.225 (0.938, 1.599)	0.410, 3.2%	No	0.592, 0.315	Moderate
Alpha-fetoprotein (High vs. Low)	10 (1,612)	1.806 (0.821, 3.972)	0.000, 85.1%^#^	No	0.929,0.637	Low
Tumor grade (III–IV/I–II)	15 (2,109)	1.653 (1.201, 2.276)*	0.039, 43.1%	Yes	0.347, 0.241	Moderate
Microvascular invasion (Present/Absent)	9 (866)	1.830 (1.009, 3.318)*	0.011, 59.7%^#^	Yes	0.754,0.420	Low
Child–Pugh score (B–C vs. A)	4 (616)	1.254 (0.414,3.798)	0.147, 44.0%	No	0.308,0.271	Low
Differentiation(H vs. W–M)	9 (979)	0.742 (0.264, 2.091)	0.000, 79.9%^#^	No	0.917,0.626	Low

*Significant differences; ^#^Substantial heterogeneity

Our systematic review and meta-analysis followed PRISMA checklist (22, 23) and was registered with PROSPERO website (24). The review by Yu et al. presented an idea that GPC-3 is a new HCC biomarker discovered after AFP, which is expressed not only in tissue but also in serum. GPC-3 has high sensitivity and specificity in the diagnosis of HCC, especially in the early stage of tumorigenesis. It has great clinical application value and brings hope for the diagnosis of early HCC (73). In addition, GPC-3 combined with AFP could use as a novel risk scoring model for predicting early recurrence of HCC after curative resection, which were shown to be effective at predicting early recurrence of HCC after curative resection (74). The research study by Zhou et al. showed that diagnosis of sarcomatoid HCC is rare and has a relatively poor prognosis. A panel of markers HSP70, GS, and GPC-3 served as an independent prognostic factor for sarcomatoid HCC (75). The research study by Kaseb et al. showed that a greater GPC-3 expression is associated with a worse HCC prognosis and may be a promising prognostic marker (76). The results in the study by Miura et al. revealed that the preoperative GPC-3 levels in patients with recurrence were significantly higher than those in patients without recurrence, suggesting that GPC-3 could be a better predictive marker of risk of recurrence than AFP, and the validation of GPC-3 as a predictive marker of HCC recurrence in a larger population is warranted (77). The above studies support our results, both in terms of diagnosis and prognosis.

Our results have been confirmed not only in clinical research but also in basic experimental research. The experiment by Aydin et al. showed that the expression of p62 and GPC-3 was significantly increased in HCC tissues compared with adjacent cirrhotic liver, and GPC-3–positive exosomes can be used for HCC detection and prediction of treatment outcomes (78). Moreover, data from the study by Montalbano reveal new aspects of the role of GPC-3 in early hepatocyte transformation. In addition, we concluded that GPC-3 may serve as a new HCC immune-therapeutic target (79). All the above studies confirmed that GPC-3 should be a valuable tumor biomarker in HCC diagnosis and prognosis.

None of the previous publications related to this topic have pooled diagnostic and prognostic data (19–21). Therefore, our study shows that GPC-3 can be used as a marker for the diagnosis and prognosis of HCC more effectively. GPC-3 belongs to heparin sulfate protein polysaccharide family and anchors to cell surface by glycosylphosphatidylinositol. Glypicans interact with growth factors and play significant roles in cell proliferation, differentiation, and migration (80, 81). GPC-3 could be used as a biomarker, in which GPC-3 gene could be a potential target for promoting hepatoma cell apoptosis and inhibiting metastasis through the Wnt/β-catenin and Hedgehog signaling pathways (80–82). The expression of GPC-3 is related with tumor size of HCC, which suggests that GPC-3 may potentially become an early diagnostic biomarker of HCC. Mechanism research has suggested that the accuracy and sensitivity for early diagnosis of HCC by using combined serum GPC-3 and AFP were better than AFP alone. Therefore, the above mechanisms and *in vivo* studies confirm our results.

There are also some limitations among our included studies. First, most of our included original research studies were from China, which may cause geographic heterogeneity. Moreover, cutoff values of GPC-3 in all studies were inconsistent, which may also lead to the heterogeneity among our studies (**Tables 1**, **S1**, **S2**). Second, baseline indicators were not balanced in diagnostic accuracy research studies. The cause maybe that HCC itself has a higher incidence in men, is more likely to be accompanied by cirrhosis, and has a worse Child–Pugh grading of liver function than the control group (**Table 1**). Third, few research studies provide data of combination diagnostic value of GCP-3 + AFP/GP73. However, we still suppose that the results were credible. Interestingly, the combination of GCP-3 + AFP + GP73 got lower diagnostic value than the combination of the above two, probably because only two articles provided the original data (**Figure 2**; **Table 2**). Last but not least, there was no combined prognostic data of GCP-3 and AFP to evaluate its combined prognostic value (**Figure 3**; **Table 3**), which did not correspond with the diagnostic studies.

In conclusion, our results suggested that GCP-3 could be used as a useful biomarker in HCC diagnosis and prognosis, especially in evaluated diagnostic value in combination with AFP or GP73, and in forecasting worse survival data of overexpression GPC-3. Therefore, we recommend that patients with cirrhosis should frequently diagnose whether there is HCC with detection by GPC-3 + AFP. Furthermore, for patients with HCC, if there was a GPC-3 overexpression, then the tumor can easily invade and affect survival. However, the conclusion still needs to be confirmed in large-scale diagnostic and prognostic research studies of GPC-3.

## Data availability statement

The original contributions presented in the study are included in the article/**Supplementary Material**. Further inquiries can be directed to the corresponding author.

## Author contributions

DJ: Conceptualization, Methodology, Formal analysis, Data Curation, Resources, Writing- Original draft preparation. YZ: Data curation, Visualization, Investigation, Writing- Original draft preparation and Reviewing and Editing. YW: Data curation, Visualization, Investigation. FX: Software, Validation, Supervision. JL: Formal analysis, Software, Validation. WW: Conceptualization, Data curation, Methodology, Writing- Reviewing and Editing.

## Conflict of interest

The authors declare that the research was conducted in the absence of any commercial or financial relationships that could be construed as a potential conflict of interest.

## Publisher’s note

All claims expressed in this article are solely those of the authors and do not necessarily represent those of their affiliated organizations, or those of the publisher, the editors and the reviewers. Any product that may be evaluated in this article, or claim that may be made by its manufacturer, is not guaranteed or endorsed by the publisher.
