# Supplementary figures and images for: Diagnostic accuracy and prognostic significance of Glypican-3 in hepatocellular carcinoma: A systematic review and meta-analysis
Source: Front Oncol. 2022 Sep 23;12:1012418. doi: 10.3389/fonc.2022.1012418 (PMC9539414; doi:10.3389/fonc.2022.1012418)

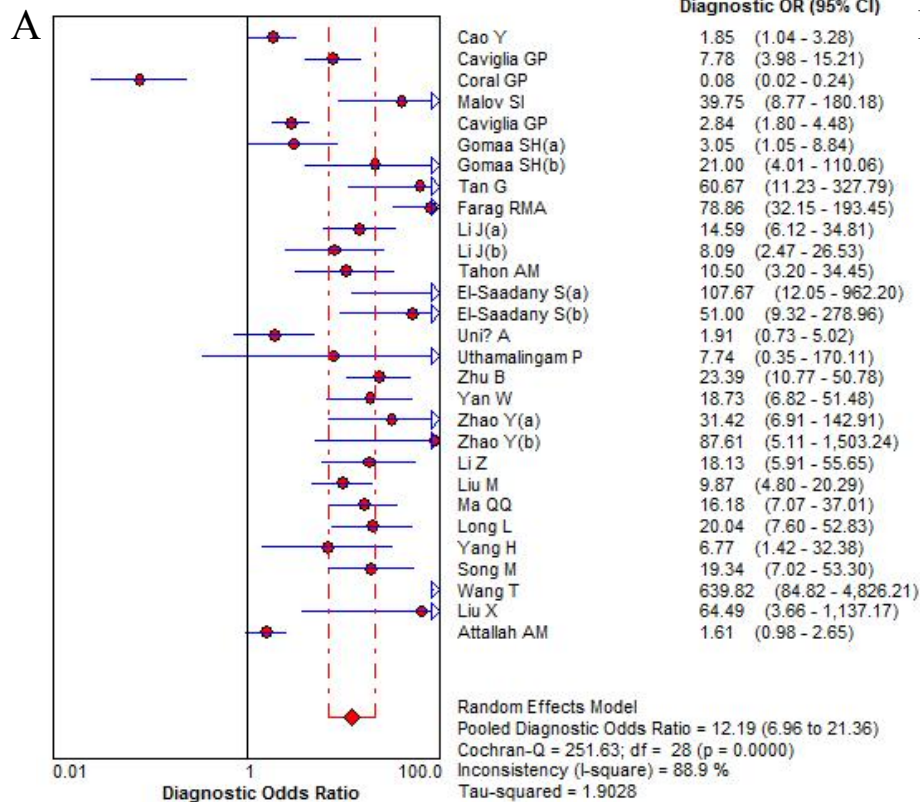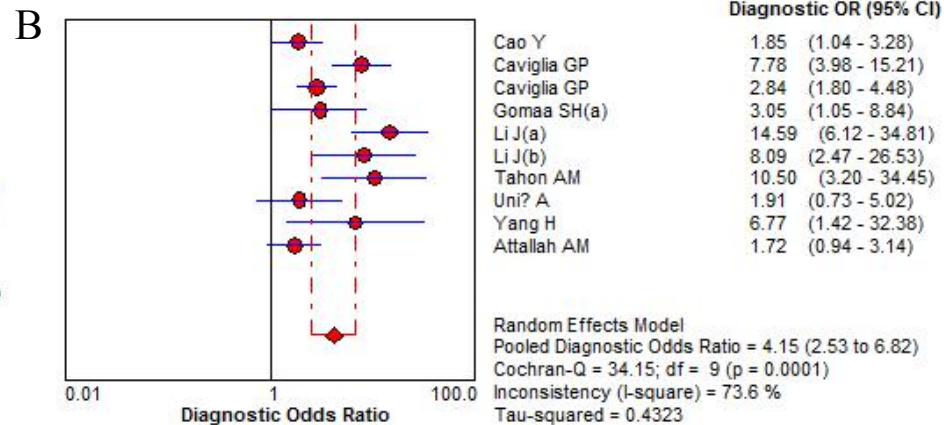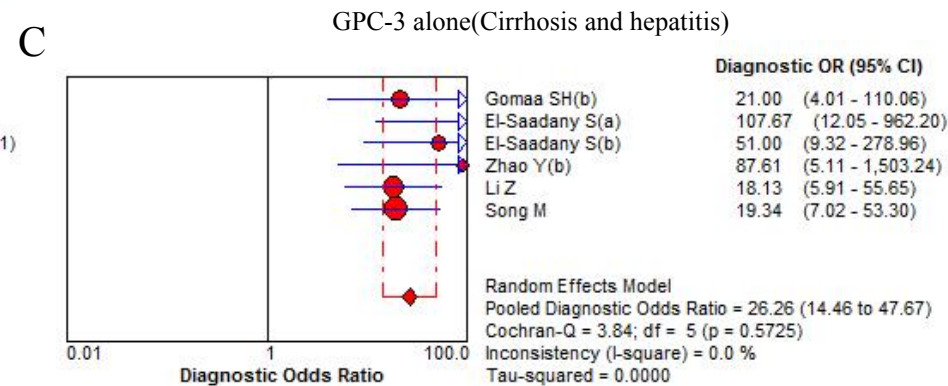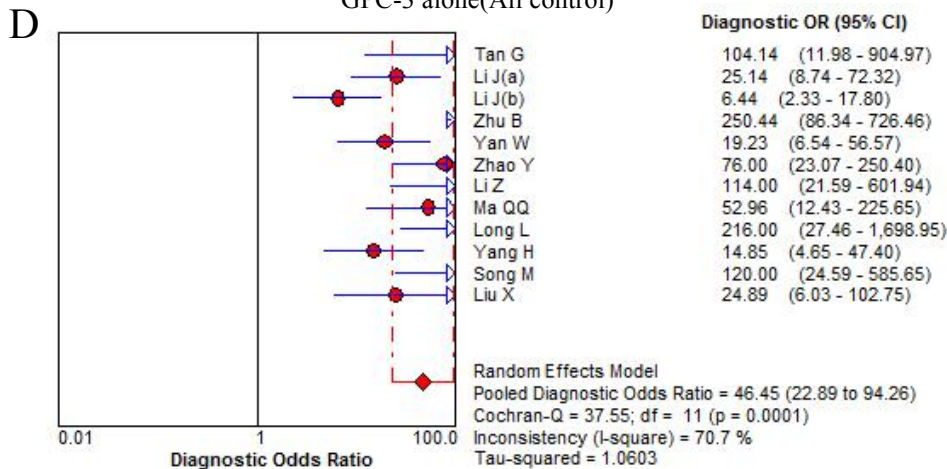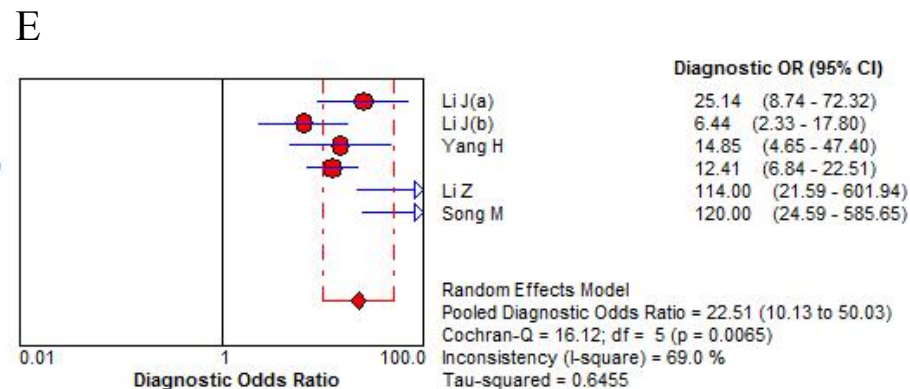

GPC-3+AFP (All control)

GPC-3+GP73 (All control)

Supplement: Supplementary file 1 [file DataSheet_1.pdf]
