# Supplementary material for: Diagnostic accuracy and prognostic significance of Glypican-3 in hepatocellular carcinoma: A systematic review and meta-analysis
Source: Front Oncol. 2022 Sep 23;12:1012418. doi: 10.3389/fonc.2022.1012418 (PMC9539414; doi:10.3389/fonc.2022.1012418)
Supplement: Supplementary file 2 [file Table_1.doc]

**Table S1 Baseline characteristic of included diagnosis research**

| **Study(year)** | **Region** | **Sample type** | **Control type** | **Detect method** | **Cut-off value** | **Sample size(Gender)** | **Age** | **HBV/HCV** | **Cirrhosis** | **Child-Pugh score** | **TP** | **FN** | **TN** | **FP** |
| --- | --- | --- | --- | --- | --- | --- | --- | --- | --- | --- | --- | --- | --- | --- |
| Cao Y,2021[1] | China | S:HCC | C:LC+HC | [Serum](javascript:;), ELISA | >5 ng/mL | S:96  C(a):68  C(b):36 | S:54.68±7.35  C(a):54.89±7.56  C(b):54.59±7.48 |  |  |  | 64 | 32 | 50 | 54 |
| Caviglia GP,2021[2] | Italy | S:HCC | C:LC | [Serum](javascript:;), ELISA | >64pg/mL | S:72(57/15)  C:119(58/61) | S:67 (62–70)  C:58 (49–66) |  | S:63/9  C:66/53 |  | 45 | 27 | 98 | 21 |
| Coral GP,2021[3] | Brasil | S:HCC | C:Non-HCC | [Serum](javascript:;),IHC |  | S:57  C:99 |  |  |  |  | 37 | 20 | 4 | 95 |
| Malov SI,2021[4] | Russia | S:HCC | C:HCV | [Serum](javascript:;), ELISA | 0.057ng/mL | S:55(38/17)  C:55(35/20) | S:59.9±4.5  C:57.7±10.5 |  |  | S:44/11  C:42/13 | 33 | 22 | 53 | 2 |
| Caviglia GP,2020[8] | Italy | S:HCC | C:LC | [Serum](javascript:;), CLEIA | >73 pg/mL | S:149(123/26)  C:200(134/66) | S:67 (31–89)  C:61 (33–82) | S:40/109-109/40  C:52/148-148/52 |  | S:18/131  C:12/188 | 109 | 40 | 102 | 98 |
| Gomaa SH,2020[9] | Egypt | S:HCC | C(a):HCV  C(b):HC |  |  | S:30(20/10)  C(a):30(12/18)  C(b):20(10/10) | S:60.9 ± 9.66  C(a):56.7 ± 8.7  C(b):42.9 ± 6.54 |  |  | S:29/1  C:26/4 | 17/21 | 13/9 | 21/18 | 9/2 |
| Tan G,2020[10] | China | S:HCC | C:Non-HCC | [Serum](javascript:;), ELISA | 498.7pg/mL | S:34(30/4)  C:28(20/8) | S:52.8±11.5  C:53.0±12.6 |  |  |  | 28 | 6 | 26 | 2 |
| Farag RMA,2019[11] |  | S:HCC | C:Non-HCC |  | 4.9pg/mL | S:145(138/7)  C:105(70/30) |  | S:20/105-105/20  C:27/48-37/38 | S:125/20  C:75/30 |  | 138 | 7 | 84 | 21 |
| Li J,2019[12] | China | S:HCC | C(a):LC+HCV  C(b):LC | [Serum](javascript:;), ELISA | 0.850pg/mL | S:47(31/16)  C(a):54(31/23)(HCV)  C(b):35(22/13)(LC) | S:63±10  C(a):37±12  C(b):55±8 |  |  |  | 37/24 | 10/23 | 71/31 | 18/4 |
| Tahon AM,2019[13] | Egypt | S:HCC | C:LC | [Serum](javascript:;), ELISA | 6.595pg/mL | S:40(33/7)  C:30(23/7) | S:61.25 ± 4.17  C:53.03 ± 4.63 | S:2/38  C:3/27 |  |  | 35 | 5 | 18 | 12 |
| El-Saadany S,2018[14] | Egypt | S(a):HCC(AFP Less than 400ug/l )  S(B):HCC(AFP more than 400ug/l ) | C:HC | [Serum](javascript:;), ELISA |  | S(a):40(15/25)  S(b):40(23/17)  C:20(10/10) | S(a):57.93±11.14  S(b):57.38±8.58  C:44.28±12.01 |  |  |  | 34/34 | 6/6 | 19/18 | 1/2 |
| Unić A,2018[15] |  | S:HCC | C:LC | [Serum](javascript:;), ELISA | 5.8μg/L | S:40(33/7)  C:30(23/7) | S:67 (53–79)  C:64 (42–81) |  |  |  | 21 | 19 | 19 | 11 |
| Uthamalingam P,2018[16] | Chandigarh | S:HCC | C:Non-HCC | IHC |  | S:12  C:10 |  |  |  |  | 3 | 9 | 10 | 0 |
| Zhu B,2016[20] | China | S:HCC | C:Non-HCC | [Serum](javascript:;), ELISA |  | S:107  C:154 |  |  |  |  | 63 | 44 | 147 | 9 |
| Yan W,2015[24] | China | S:HCC | C:Non-HCC | IHC | 400ng/L | S:34  C:82 |  |  |  |  | 27 | 7 | 68 | 14 |
| Zhao Y,2015[25] | China | S:HCC | C(a):digestive system disorders  C(b):HC | [Serum](javascript:;), ELISA | 8.98 μg/L | S:50(36/14)  C(a):60(40/20)  C(b):40(22/18) | S:56(32-81)  C(a):57(36-81)  C(b):54(35-80) |  |  |  | 26 | 24 | 58/40 | 2/0 |
| Li Z,2014[26] | China | S:HCC | C:HC | [Serum](javascript:;), ELISA | 6.08pg/mL | S:42(29/13)  C:40 | S:49.9(35-69)  C:(26-45) |  |  |  | 32 | 10 | 34 | 6 |
| Liu M,2014[27] | China | S:HCC | C:Non-HCC | IHC | 25% | S:102(52/50)  C:80(40/40) | S:60.3±6.5  C:59.8±7.6 |  |  |  | 67 | 35 | 67 | 13 |
| Ma QQ,2014[28] | China | S:HCC | C:Non-HCC | [Serum](javascript:;), ELISA | 60pg/mL | S:54(33/21)  C:164(97/67) |  |  |  |  | 46 | 8 | 121 | 43 |
| Long L,2013[31] | China | S:HCC | C:Non-HCC | [Serum](javascript:;), ELISA | 805.38 pg/mL | S:43  C:129 |  |  |  |  | 23 | 20 | 122 | 7 |
| Yang H,2013[32] | China | S:HCC | C:HBV | [Serum](javascript:;), WB | 15ng/mL | S:45(33/12)  C:32(24/8) | S:51.8±10.3  C:50.0±12.9 |  |  |  | 14 | 31 | 30 | 2 |
| Song M,2011[36] | China | S:HCC | C:HC | ELISA,IHC | 120 ng/ml | S:57(47/10)  C:47 | S:48.6  C:- |  |  |  | 44 | 13 | 40 | 7 |
| Wang T,2011[37] | China | S:HCC | C:Non-HCC | IHC | >3 score | S:114  C:154 |  |  |  |  | 92 | 22 | 153 | 1 |
| Liu X,2009[38] | China | S:HCC | C:Non-HCC | WB |  | S:35(19/16)  C:30(19/11) |  |  |  |  | 18 | 17 | 30 | 0 |

24个
