# Supplementary material for: Diagnostic accuracy and prognostic significance of Glypican-3 in hepatocellular carcinoma: A systematic review and meta-analysis
Source: Front Oncol. 2022 Sep 23;12:1012418. doi: 10.3389/fonc.2022.1012418 (PMC9539414; doi:10.3389/fonc.2022.1012418)
Supplement: Supplementary file 3 [file Table_2.doc]

**Table S2 Baseline characteristic of included prognosis research**

| **Study(year)** | **Region** | **Detect method** | **Cut-off value** | **Sample size(Gender)** | **Age(Yong vs old)** | **Tumor size (Small vs Big)** | **HBV(Y/N)** | **HCV(Y/N)** | **Cirrhosis(Y/N)** | **Alpha-fetoprotein(<20 ng/mL/>20 ng/mL )** | **Tumor grade(I-II/III-IV)** | **Microvascular invasion(Y/N)** | **Child-Pugh score (A vs B-C)** | **BCLCgrade(A-B vs C-D)** | **Differentiation(W-M vs H)** | **Overall survival rate(H vs L)** | **Disease-free survival(H vs L)** |
| --- | --- | --- | --- | --- | --- | --- | --- | --- | --- | --- | --- | --- | --- | --- | --- | --- | --- |
| Wang J,2021[5] | China |  |  | H:251  L:113 |  |  |  |  |  |  |  |  |  |  |  | 1.26(0.66,2.43) |  |
| Zhao J,2021[6] | China | Immunoreactivity | ≥ 5% | H:100(83/17)  L:43(39/4) | H:56.8 ± 10.2  L:59.1 ± 11.3 | H:3.96 ± 1.92  L:4.02 ± 1.52 | H:97/3  L:39/4 |  |  | H:26/74  L:34/9 | H:24/76  L:10/33 | H:44/56  L:10/33 |  |  |  |  |  |
| Zhou X,2021[7] | China | [Serum](javascript:;),qRT⁃PCR | 1/3 | H:97(75/23)  L:29(22/6) | H:50/48  L:12/16 | H:37/61  L:14/14 |  |  |  |  |  |  |  | H:71/27  L:21/7 | H:56/42  L:8/24 |  |  |
| Xue R, 2017[17] | China | IHC | >30% | H:241(208/33)  L:75(60/15) | H:53.08± 11.89  L:54.89± 11.33 |  | H:198/43  L:57/18 |  | H:216/25  L:67/8 | H:150/91  L:68/7 | H:135/106  L:52/23 |  |  |  |  |  |  |
| Jeon Y, 2016[18] | Korea | IHC | >5% | H:153(111/42)  L:32(27/5) | H:54.4± 11.5  L:59.4± 8.9 | H:115/38  L:24/8 | H:122/31  L:23/9 | H:12/141  L:3/29 | H:85/68  L:20/12 | H:82/71  L:7/25 | H:127/26  L:29/3 | H:74/79  L:7/25 |  |  |  | 1.0 (0.39, 2.58) | 1.85 (0.83, 4.12) |
| Wang L, 2016[19] | China | IHC | >33% | H:66  L:69 |  |  | H:50/16  L:50/19 |  | H:44/22  L:45/24 | H:43/23  L:46/23 | H:26/44  L:29/40 |  |  |  | H:3/63  L:2/1 | 4.259(2.03,8.934) |  |
| Cui X, 2015[21] | China | IHC | >10% | H:74(65/9)  L:30(28/2) | H:50±8  L:48±9 |  |  |  |  |  | H:28/46  L:20/10 | H:33/41  L:7/23 |  |  | H:59/15  L:29/1 | 2.691(1.097,6.605) | 2.125(1.010,4.474) |
| Haruyama Y, 2015[22] | Japan | Serum, Immunohistochemistry (IHC) | >2 score | H:69(51/18)  L:46(40/6) | H:16/53  L:11/35 | H:48/21  L:34/12 | H:14/55  L:12/34 | H:33/36  L:34/12 | H:39/30  L:28/28 |  | H:37/32  L:25/21 | H:32/37  L:19/27 | H:58/11  L:45/1 |  |  | 2.307(1.002,5.310) | 3.347(1.343,8.339) |
| Pan C, 2015[23] | China | IHC |  | H:270(237/33)  L:30(26/4) | H:132/138  L:11/19 | H:135/135  L:10/20 | H:229/38  L:22/7 |  | H:179/91  L:23/7 | H:94/174  L:10/19 | H:236/34  L:23/7 | H:158/112  L:26/4 | H:246/24  L:28/2 |  | H:269/1  L:29/1 | 1.634(1.001,2.667) | 1.597(1.032,2.469) |
| Liu M,2014[27] | China | IHC |  | H:67  L:35 |  | H:18/49  L:24/11 |  |  |  |  |  | H:28/39  L:6/29 |  |  | H:49/18  L:10/25 |  |  |
| Fan G,2013[29] | China | IHC | >9 score | H:28(17/11)  L:7(4/3) | H:7/19  L:2/6 | H:15/12  L:6/2 |  |  |  |  | H:15/9  L:6/1 |  |  |  | H:13/10  L:8/5 |  |  |
| Fu SJ,2013[30] | China | IHC |  | H:109(95/14)  L:51(45/6) | H:49/60  L:28/23 | H:30/79  L:20/31 | H:97/12  L:40/11 |  | H:87/22  L:38/13 |  | H:56/53  L:38/13 |  | H:104/5  L:47/4 |  | H:81/28  L:42/9 |  |  |
| Ning S,2012[33] | China | IHC | >6 score | H:32(29/3)  L:29(26/3) | H:20/12  L:16/13 | H:12/20  L:12/17 | H:26/6  L:27/2 |  | H:28/20  L:12/17 | H:13/19  L:16/13 | H:20/12  L:25/4 |  |  |  | H:20/12  L:25/4 | 1.905(1.063,3.415) |  |
| Wang YL,2012[34] | China | IHC | >10% |  |  | H:17/3  L:9/2 |  |  |  | H:6/14  L:5/6 | H:6/14  L:6/5 | H:14/6  L:2/9 |  |  | H:18/2  L:7/4 |  |  |
| Yu MC,2012[35] | China | IHC | >30% | H:241(208/33)  L:75(60/15) | H:53.08± 11.89  L:54.89± 11.33 |  | H:216/25  L:67/8 |  | H:198/43  L:57/18 | H:150/91  L:68/7 | H:135/106  L:52/23 |  |  |  |  |  |  |
| Wang T,2011[37] | China | IHC | >3 score | H:92(76/16)  L:22(18/4) | H:48/40  L:5/7 |  | H:68/24  L:12/10 |  | H:83/9  L:16/6 | H:38/34  L:11/4 | H:58/34  L:16/8 | H:18/74  L:4/18 |  |  |  |  |  |
| Li B,2006[39] | China | WB |  | H:33(20/13)  L:8(7/1) | H:14/19  L:5/3 | H:8/25  L:2/6 | H:21/12  L:6/2 |  | H:29/4  L:6/2 | H:18/15  L:2/6 | H:11/22  L:6/2 | H:14/19  L:7/1 | H:27/6  L:6/2 |  |  |  |  |
| Ding G,2005[40] | China | IHC |  | H:29(27/2)  L:12(11/1) | H:16/13  L:9/3 | H:5/23  L:7/6 | H:26/3  L:11/1 |  |  |  | H:3/26  L:4/8 | H:5/24  L:2/10 |  |  |  |  |  |

CHIDE分级，分化

18个
