# Supplementary material for: Diagnostic accuracy and prognostic significance of Glypican-3 in hepatocellular carcinoma: A systematic review and meta-analysis
Source: Front Oncol. 2022 Sep 23;12:1012418. doi: 10.3389/fonc.2022.1012418 (PMC9539414; doi:10.3389/fonc.2022.1012418)
Supplement: Supplementary file 4 [file Table_3.doc]

**Table S3 Quality assessment of included of studies by QUADAS-2 scales.**

| Study | PATIENT SELECTION | | | | INDEX TEST(S) | | | REFERENCE STANDARD | | | FLOW AND TIMING | | | | |
| --- | --- | --- | --- | --- | --- | --- | --- | --- | --- | --- | --- | --- | --- | --- | --- |
|  | Was a consecutive or random sample of patients enrolled? | Was a case-control design avoided? | Did the study avoid inappropriate exclusions? | **Is there concern that the included patients do not match**  **the review question?** | Were the index test results interpreted without  knowledge of the results of the reference standard? | If a threshold was used, was it pre-specified? | **Is there concern that the index test, its conduct, or**  **interpretation differ from the review question?** | Is the reference standard likely to correctly classify the target  condition? | Were the reference standard results interpreted without  knowledge of the results of the index test? | **Is there concern that the target condition as defined by**  **the reference standard does not match the review**  **question?** | Was there an appropriate interval between index test(s)  and reference standard? | Did all patients receive a reference standard? | Did patients receive the same reference standard? | Were all patients included in the analysis? | **Could the patient flow have introduced bias?** |
| Zhu M (2020)[36模板 | Yes | Yes | Unclear | Unclear | Yes | Unclear | Unclear | Yes | Unclear | Unclear | Unclear | Yes | Yes | Yes | Unclear |
| Cao Y,2021[1] |  |  |  |  |  |  |  |  |  |  |  |  |  |  |  |
| Caviglia GP,2021[2] |  |  |  |  |  |  |  |  |  |  |  |  |  |  |  |
| Coral GP,2021[3] |  |  |  |  |  |  |  |  |  |  |  |  |  |  |  |
| Malov SI,2021[4] |  |  |  |  |  |  |  |  |  |  |  |  |  |  |  |
| Caviglia GP,2020[8] |  |  |  |  |  |  |  |  |  |  |  |  |  |  |  |
| Gomaa SH,2020[9] |  |  |  |  |  |  |  |  |  |  |  |  |  |  |  |
| Tan G,2020[10] |  |  |  |  |  |  |  |  |  |  |  |  |  |  |  |
| Farag RMA,2019[11] |  |  |  |  |  |  |  |  |  |  |  |  |  |  |  |
| Li J,2019[12] |  |  |  |  |  |  |  |  |  |  |  |  |  |  |  |
| Tahon AM,2019[13] |  |  |  |  |  |  |  |  |  |  |  |  |  |  |  |
| El-Saadany S,2018[14] |  |  |  |  |  |  |  |  |  |  |  |  |  |  |  |
| Unić A,2018[15] |  |  |  |  |  |  |  |  |  |  |  |  |  |  |  |
| Uthamalingam P,2018[16] |  |  |  |  |  |  |  |  |  |  |  |  |  |  |  |
| Zhu B,2016[20] |  |  |  |  |  |  |  |  |  |  |  |  |  |  |  |
| Yan W,2015[24] |  |  |  |  |  |  |  |  |  |  |  |  |  |  |  |
| Zhao Y,2015[25] |  |  |  |  |  |  |  |  |  |  |  |  |  |  |  |
| Li Z,2014[26] |  |  |  |  |  |  |  |  |  |  |  |  |  |  |  |
| Liu M,2014[27] |  |  |  |  |  |  |  |  |  |  |  |  |  |  |  |
| Ma QQ,2014[28] |  |  |  |  |  |  |  |  |  |  |  |  |  |  |  |
| Long L,2013[31] |  |  |  |  |  |  |  |  |  |  |  |  |  |  |  |
| Yang H,2013[32] |  |  |  |  |  |  |  |  |  |  |  |  |  |  |  |
| Song M,2011[36] |  |  |  |  |  |  |  |  |  |  |  |  |  |  |  |
| Wang T,2011[37] |  |  |  |  |  |  |  |  |  |  |  |  |  |  |  |
| Liu X,2009[38] |  |  |  |  |  |  |  |  |  |  |  |  |  |  |  |
