# Supplementary material for: Diagnostic accuracy and prognostic significance of Glypican-3 in hepatocellular carcinoma: A systematic review and meta-analysis
Source: Front Oncol. 2022 Sep 23;12:1012418. doi: 10.3389/fonc.2022.1012418 (PMC9539414; doi:10.3389/fonc.2022.1012418)
Supplement: Supplementary file 5 [file Table_4.doc]

**Table S4 Risk of Bias Assessment using the Newcastle-Ottawa Scale for Cohort studies.**

| **Publication(year)** | **Representativeness of the Exposed Cohort** | **Selection of** the Non-Exposed Cohort | **Ascertainment of Exposure** | **Demonstration That Outcome of Interest Was Not Present at Start of Study** | **Comparability of Cohorts on the Basis of the Design or Analysis** | **Assessment of Ou** tcome | **Was Follow-Up Long Enough for Outcomes to Occur** | **Adequacy of Follow Up of Cohorts** | **Overall rating and TOTAL SCORE / 10** |
| --- | --- | --- | --- | --- | --- | --- | --- | --- | --- |
|  |  |  |  |  | **(/2)** |  |  |  |  |
| Rong W(2019)[37]模板 | 1 | 0 | 1 | 1 | 1 | 1 | 1 | 1 | 7 |
| Wang J,2021[5] |  |  |  |  |  |  |  |  |  |
| Zhao J,2021[6] |  |  |  |  |  |  |  |  |  |
| Zhou X,2021[7] |  |  |  |  |  |  |  |  |  |
| Xue R, 2017[17] |  |  |  |  |  |  |  |  |  |
| Jeon Y, 2016[18] |  |  |  |  |  |  |  |  |  |
| Wang L, 2016[19] |  |  |  |  |  |  |  |  |  |
| Cui X, 2015[21] |  |  |  |  |  |  |  |  |  |
| Haruyama Y, 2015[22] |  |  |  |  |  |  |  |  |  |
| Pan C, 2015[23] |  |  |  |  |  |  |  |  |  |
| Liu M,2014[27] |  |  |  |  |  |  |  |  |  |
| Fan G,2013[29] |  |  |  |  |  |  |  |  |  |
| Fu SJ,2013[30] |  |  |  |  |  |  |  |  |  |
| Ning S,2012[33] |  |  |  |  |  |  |  |  |  |
| Wang YL,2012[34] |  |  |  |  |  |  |  |  |  |
| Yu MC,2012[35] |  |  |  |  |  |  |  |  |  |
| Wang T,2011[37] |  |  |  |  |  |  |  |  |  |
| Li B,2006[39] |  |  |  |  |  |  |  |  |  |
| Ding G,2005[40] |  |  |  |  |  |  |  |  |  |
