# Supplementary material for: Diagnostic accuracy and prognostic significance of Glypican-3 in hepatocellular carcinoma: A systematic review and meta-analysis
Source: Front Oncol. 2022 Sep 23;12:1012418. doi: 10.3389/fonc.2022.1012418 (PMC9539414; doi:10.3389/fonc.2022.1012418)
Supplement: Supplementary file 6 [file Table_5.doc]

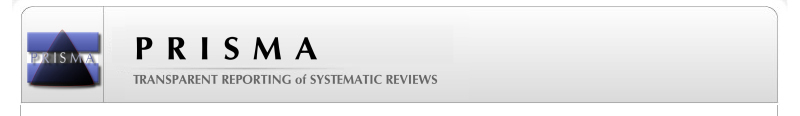
**PRISMA 2009 Flow Diagram**

**Screening**

**Included**

**Eligibility**

**Identification**

Records identified through database searching
(n = 2508)

Additional records identified through other sources
(n = 0 )

Records after duplicates removed
(n =931)

Records screened
(n =543)

Records excluded
(n = 454)

Full-text articles assessed for eligibility
(n = 89)

Full-text articles excluded, with reasons
(n = 49)

Studies included in qualitative synthesis
(n = 40)

Studies included in quantitative synthesis (meta-analysis)
(n = 40)
